# Preparation and in vivo and ex vivo studies of sirolimus nano-in-situ gel ophthalmic formulation

**DOI:** 10.1186/s12951-024-02668-1

**Published:** 2024-07-16

**Authors:** Ye Liu, Xu Chen, Xinghao Chen, Jie Chen, Han Zhang, Haonan Xu, Lu Jin, Qiao Wang, Zhan Tang

**Affiliations:** 1 School of Pharmacy, Hangzhou Medical College, Hangzhou, 310013 China; 2 Key Laboratory of Neuropsychiatric Drug Research of Zhejiang Province, Hangzhou Medical College, Hangzhou, 310013 China

**Keywords:** Sirolimus, Ionomer in situ gel, Gellan gum, Pharmacokinetics, Corneal neovascularization

## Abstract

Sirolimus (SR) is a macrolide with antifungal and antitumor immunosuppressant properties, classified as a selective inhibitor of mammalian target of rapamycin (mTOR). In this study, an ionic in situ gel of SR (SR-SUS-ISG) was formulated using gellan gum, exhibiting stability regardless of temperature and pH variations, causing minimal irritation. Harnessing the physiological conditions of the eye, SR-SUS-ISG underwent gelation upon contact with ions, increasing drug viscosity and prolonging retention on the ocular surface. Concurrently, SR-SUS-ISG displayed favorable shear dilution properties, reducing viscosity at ambient temperature, enhancing fluidity, and facilitating convenient packaging and transport. Biocompatibility assessments on both human corneal epithelial cells and rabbit eyes demonstrated that SR-SUS-ISG could well be tolerated. Pharmacokinetic investigations in rabbit ocular aqueous humor revealed sustained release, improved corneal penetration, and enhanced bioavailability. Additionally, in a rat corneal alkali burn model, SR-SUS-ISG exhibited inhibitory effects on corneal neovascularization, associated with decreased levels of the inflammatory factors VEGF and MMPs. These findings suggested that SR-SUS-ISG held promise as an effective ocular drug delivery system.

**Graphical Abstract:**

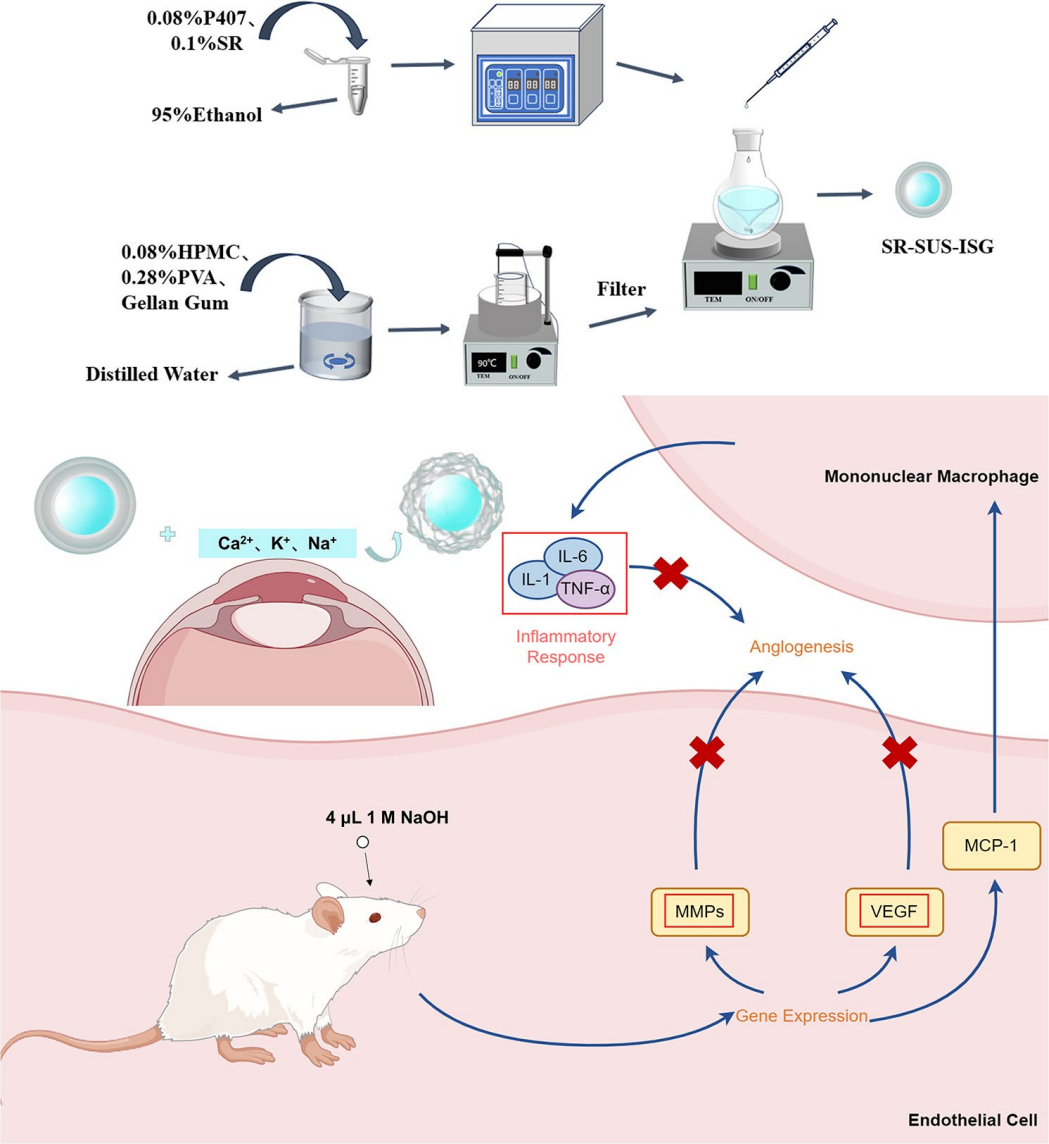

## Introduction

**Fig. 1:**
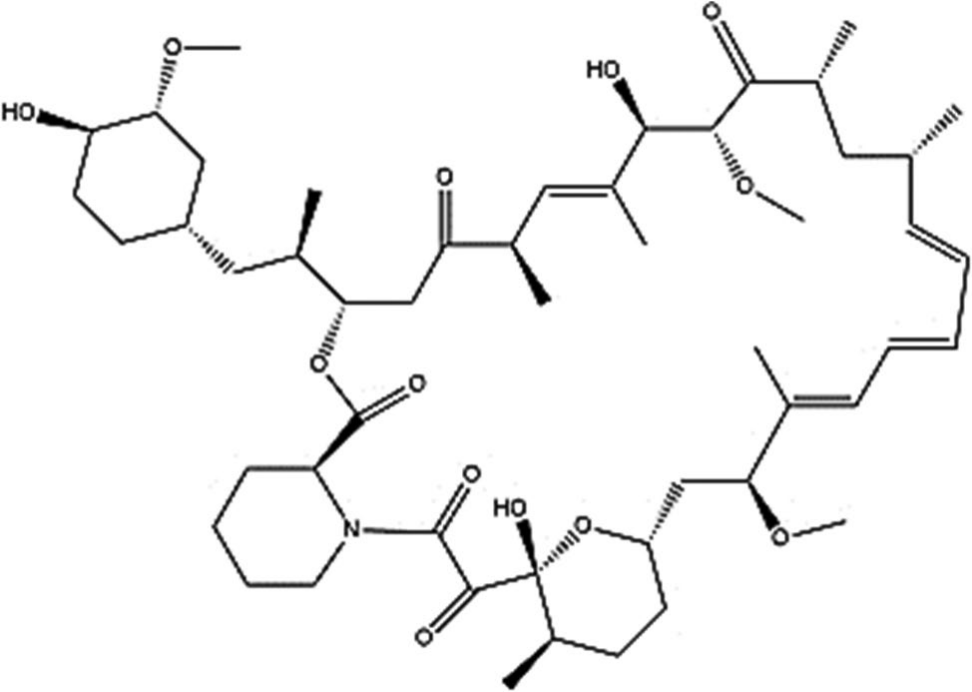
The structural formula of Sirolimus [5] (Copyright 2023, Drug Delivery)

Sirolimus (SR), also known as rapamycin (RAPA), is a macrolide antifungal and antitumor drug that falls under the category of immunosuppressants [1]. Its structural formula is depicted in Fig. 1. Functioning as a selective inhibitor of mammalian target of rapamycin (mTOR), a critical serine-threonine kinase often upregulates in certain human tumors, SR forms an immunosuppressive complex with the intracellular protein FKBP12. This complex prevents the activation of mammalian mTOR [2]. Consequently, SR hinders the progression of the cell cycle from the G1 to the S phase, leading to the inhibition of T-lymphocyte activation, proliferation, and antibody production [3]. Additionally, it acts by suppressing the release of inflammatory cytokines [4].

Initially approved for use as an immunosuppressant in kidney transplantation [6], SR received subsequent approval from the U.S. Food and Drug Administration (FDA) in 2007 for the treatment of advanced kidney cancer. Several studies have highlighted the potential of SR in treating ocular diseases and effectively preventing glaucomatous neurodegeneration [7]. This effect is achieved through the inhibition of mTOR, attenuation of the HIF-1α-VEGF pathway, and accompanied by the downregulation of apoptotic Caspase-3 [8]. In addition, SR has been found to protect tear secretion and the ocular surface from dry eye by alleviating endoplasmic reticulum (ER) stress-induced vascular injury and inflammation of the lacrimal gland and ocular surface [9].

**Fig. 2:**
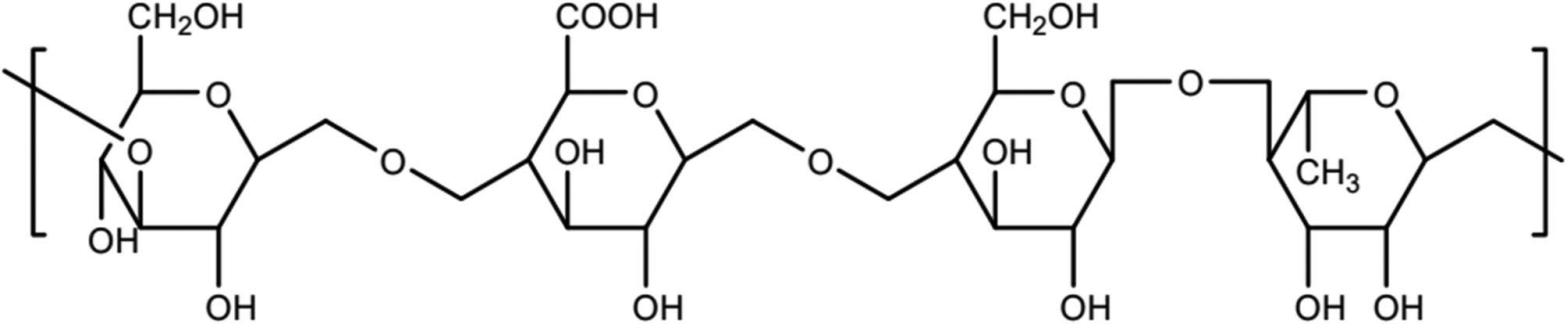
The structural formula of gellan gum [27] (Copyright 2022, Polymers)

There are currently no marketed ophthalmic formulations of Sirolimus. Systemic administration of SR is associated with adverse effects such as nephrotoxicity, neurotoxicity, hyperglycemia, and joint pain. To address these issues, the development of ophthalmic formulations for topical application has been explored as a potential solution for treating ocular disorders while minimizing systemic adverse effects. SR presents challenges in formulation due to its extreme hydrophobic nature (solubility of 2.6 µg/mL) [10] and high molecular weight (914.172) [11]. The absence of ionizable functional groups in solutions at pH 1 to 10 necessitates the preparation of nano-formulations, such as nano-suspensions, to enhance drug solubility. Eye drops, as a non-invasive and easily administrable method, are preferred over intravitreal injections [12–14]. However, the short residence time of conventional nanofluids on the ocular surface poses absorption challenges. To address this, nano-formulations can be combined with in situ gels to create innovative delivery formats. in situ gels undergo a phase transition at the administration site in response to factors like temperature, pH, and ionic strength, transforming from a liquid to a non-chemically cross-linked semi-solid gel [15, 16]. This characteristic allows for prolonged retention time of the drug in the eye, reduced frequency of administration, improved bioavailability, and sustained-release effects compared to ordinary eye drops, achieving the purpose of slow-release and long-lasting effects [17]. Gellan gum is a water-soluble polysaccharide produced extracellularly by *Sphingomonas elodea*, as shown in Fig. 2. Comprising four sugar molecules (D-glucose, D-glucuronic acid, D-glucose, L-rhamnose) connected by glycosidic bonds, it has a high molecular weight, and the first glucose molecule is connected by a β-1,4 glycosidic bond [18–20]. Tear fluid contains various cations, including Ca^2+^, K^+^, and Na^+^, causing gellan gum to undergo gelation upon contact [21]. Numerous studies have demonstrated the suitability of gellan gel for local administration [22–24], with several reports focusing on its use in ophthalmic formulations [25, 26].

The cornea, a transparent and non-vascularized tissue, maintains a delicate equilibrium between low levels of angiogenic factors and high levels of anti-angiogenic factors in its resting state [28]. External damage disrupts this balance, leading to increased production of angiogenic factors, resulting in corneal neovascularization. This condition leads to scarring, stromal edema, lipid deposition, and inflammation, ultimately causing corneal clarity and vision loss [29]. Current therapeutic strategies include steroids [30], anti-Vascular Endothelial Growth Factor(anti-VEGF) agents [31], gene therapy [32, 33], surgical treatment [34], and immunosuppressive agents [35, 36].

This study proposed to follow a screened sirolimus nanosuspension formulation [37] and develop ionic in situ gels with gellan gum. Given the ocular surface temperature of 34 °C and a pH range of 6.4–7.7, the gelling of ionic in situ gels was dependent on the ions in the solution, irrespective of temperature and pH. The formulation’s formulation can be adjusted to the appropriate pH and osmolality, minimizing ocular irritation. The present study aimed to assess the in vitro release behavior, biocompatibility, and antiangiogenic effects of SR-SUS-ISG through a combination of in vivo and in vitro tests.Graphical Abstract was the schematic illustration of the preparation of SR-SUS-ISG as well as the gelation process and angiogenesis inhibition process. (By Figdraw, ID: ITRSI45455)

## Materials and methods

### Materials

Sirolimus (purity ≥ 98%) was procured from Fujian Kerui Pharmaceutical Co. Hydroxypropyl Methylcellulose (HPMC) was obtained from Aladdin. Poroxam 407 (P407) was sourced from Shanghai Changwei Pharmaceutical Excipients Technology Co. Polyvinyl alcohol (PVA) was acquired from Jiangxi Alpha Hi-Tech Pharmaceuticals Co. Hypoalloylated gellan gum was purchased from Azores International Trading (Shanghai) Co. 95% ethanol (analytically pure) was obtained from Chengdu Cologne Chemical Co. Human corneal epithelial cells were sourced from Bei Na Biotechnology Co. Cell Counting Kit-8 (CCK8) was purchased from White Shark Ease. The BCA Protein Measurement Kit was acquired from CombiCentury, and RIPA Lysate was obtained from CombiCentury as well. DMSO was procured from Beyotime, coumarin-6 from Bailiwick, and Cyclosporine Eye Drops (II) from Shenyang Xingqi Ophthalmic Pharmaceutical Co. The SteadyPure RNA Extraction Kit and Evo M-MLV Reverse Transcription Premixed Kit were obtained from Acres Bioengineering Co., while the SYBR^®^ Green Pro Taq HS Premixed qPCR Kit was sourced from Acres Bioengineering Co.

### Preparation of SR-SUS-ISG

To prepare the organic phase, a mixture of 10 mg sirolimus and 8 mg Poroxam P407 was dissolved in 140 µL of 95% ethanol. In a separate process, 8 mg HPMC, 28 mg PVA, and 45 mg gellan gum were solubilized in 10 mL of ultrapure water. As shown in Graphic Abstract, after adding the excipients to the aqueous phase, they would be dissolved by heating in a water bath at 90 °C with magnet stirring. After dissolution of the excipients, the evaporated water was replenished by weighing before and after and mixed thoroughly and filtered through a 0.45 μm filter membrane into a round bottom flask when cooled to 40–50 °C. The magnetic stirrer was added for room temperature and stirred at roughly 100 rpm. The organic phase was dropwised to the centre of the stirred aqueous phase with a microsyringe. Then it was stirred for a designated duration to allow ethanol evaporation, resulting in the formation of SR-SUS-ISG. The same procedure was repeated to obtain SR-SUS, with the exception that gellan gum was omitted from the aqueous phase.

### Formulation selection and characterization

#### Formulation selection

Various concentrations of in situ gel formulations were created by incorporating gellan gum powder at concentrations of 0.35%, 0.4%, 0.45%, 0.5%, and 0.55% into the formulations. The in situ gel was blended with different ratios of artificial tears, and the viscosity was assessed at 34 °C to identify the proportions yielding maximum viscosity. Subsequently, the* in situ *gel formulations at different concentrations were evaluated at room temperature (25 °C) to conduct a screening for lower viscosity.

#### Particle size, polydispersity index, and zeta potential

The samples were aspirated at a volume of 0.1 mL and then diluted 10-fold with pure water. Subsequently, the particle size, polydispersity index (PDI), and Zeta potential of the samples were measured using a Malvern Zetasizer Nano ZS90 (Malvern Instruments Inc., Malvern, UK).

#### Content determination of SR-SUS-ISG

To determine the content, 0.1 mL of the preparation was added in 2 mL of acetonitrile and mixed well. Then 1.9 mL of 1 mM CaCl_2_ was also added (to precipitate the gellan gum). Then it was vortexed for 3–5 min, sonicated for 5 min, and centrifuged at 9000 rpm for 5 min. The supernatant was taken for high-performance liquid chromatography (HPLC) analysis (Shimadzu, LC-20AT, Japan).

#### Transmission electron microscopy

A small quantity of the sample was added dropwise onto a copper mesh. Surface moisture was absorbed using filter paper, and the sample was stained with 3% uranyl acetate. After staining, the sample was allowed to dry. Subsequently, the morphology of SR-SUS-ISG was observed using transmission electron microscopy (HT7700, Hitachi, Japan).

#### Stability study

SR-SUS-ISG were stored at 4 °C for 1, 2, 3, 4, 5, and 6 months. Particle size, PDI, Zeta potential and content were determined, respectively.

#### In vitro release

The dialysis bag (molecular weight cutoff: 14,000) was securely tied at one end using a thin string, and a volume of 0.5 mL of the preparation was added. Subsequently, the dialysis bag was tightly sealed and placed inside a vial, to which 20 mL of the release medium was added. The vial was then positioned in a constant temperature oscillator. At specified time intervals (0.25 h, 0.50 h, 0.75 h, 1 h, 2 h, 4 h, 6 h, 8 h), 1 mL of sample was withdrawn, and an equal volume of release medium was replenished. The collected samples were centrifuged at 9000 rpm and subjected to analysis using HPLC.

#### Ex vivo rabbit scleral penetration

New Zealand rabbits were humanely euthanized using air needles, and their eyeballs were carefully extracted. The sclera, evenly divided into two halves, underwent washing to eliminate excess protein from the surface. Following the addition of the release medium in the Franz diffusion cell, the sclera was placed tautly over the opening of the diffusion cell, and air was expelled. The preparation, mixed with synthetic tear fluid (STF) in a ratio of 40:7, was applied with 94 µL of the mixture at the mouth of the upper cell. Subsequently, 100 µL of the solution was withdrawn using a microsyringe and replenished with an isothermal and equal volume of release medium at 2 h, 4 h, 6 h, 8 h, 10 h, and 24 h, respectively. The collected samples were then centrifuged at 9000 rpm and subjected to analysis using HPLC.

The cumulative release rate and the cumulative permeability (Q_n_) was calculated according to Eq. (1) [38], the permeability coefficient (P_app_) was calculated according to Eq. (2) [39] and the steady-state permeation flux (J_ss_) was calculated according to Eq. (3) [40], and the cumulative permeability-time curve was plotted.

1 $${Q}_{n}=\frac{{V}_{0}{C}_{n}+ \text{V} {\sum }_{i=1}^{n-1}{C}_{i}}{{m}_{drug}}$$

V_0_ is the volume of the released medium; C_n_ is the drug concentration measured at each time point; V is the sampling volume; C_i_ is the concentration measured at the last time point; drug is the quantity of drug supplied.

2 $${P_{{\text{app}}}} = \frac{{\Delta Q}}{{\Delta t \cdot {C_0} \cdot A}}$$

3 $${{\text{J}}_{{\text{ss}}}}{\text{ = }}{{\text{P}}_{{\text{app}}}} \times {{\text{C}}_0}$$

$\frac{{\Delta Q}}{{\Delta t}}$is the change in receptor concentration (µg/h) calculated from the slope of the time-concentration curve; A is the area of the biofilm; C_0_ is the initial donor chamber concentration.

### Cells

#### Cell culture

The human corneal epithelial cell line (HCECs) was cultured in DMEM/F-12 medium supplemented with 10% fetal bovine serum and 1% penicillin/streptomycin. The cultures were maintained at a temperature of 37 °C in a humidified environment with 5% CO_2_.

#### Cell recovery

To initiate cell culture, the frozen cells were carefully removed from the liquid nitrogen tank and thawed in a 37 °C water bath. After thawing, the cells were gently centrifuged, and the resulting cell precipitate was collected. The supernatant was slowly aspirated using a pipette gun, and 5 mL of complete medium was added. The cell precipitate was then resuspended in the medium, transferred to 25 cm^2^ culture flasks, and placed in a carbon dioxide incubator for observation.

#### Cell proliferation

Upon reaching 80% confluence, HCECs were trypsinized and collected for cell counting. A 96-well plate was then inoculated with a concentration of 1 × 10^5^ cells/mL per well, with cells diluted in basal medium based on the desired concentration gradient. After 24 h of incubation, 100 µL of SR solution, Blank-SUS, Blank-SUS-ISG, SR-SUS, and SR-SUS-ISG were added to their respective wells, followed by an additional 24-hour incubation period. Subsequently, 10 µL of Cell Counting Kit-8 (CCK-8) reagent was added to each well and incubated for 2 h. The optical density (OD) value of each well in the cell culture plate was measured using a multifunctional enzyme labeler (Molecular Devices, SpectraMax iD5, USA) at a wavelength of 450 nm. The cell viability for each drug-administered group was calculated using Eq. (4):

4 $${\text{Cell viability }}\left( {\text{\% }} \right){\text{ = }}\frac{{{\text{Treated\,group}}\left( {{\text{OD}}} \right) - {\text{Blank\,group}}\left( {{\text{OD}}} \right)}}{{{\text{Control\,group}}\left( {{\text{OD}}} \right) - {\text{Blank\,group}}\left( {{\text{OD}}} \right)}}$$

#### Cell uptake

HCECs were cultured until reaching 90% confluence, then cells were digested, counted, and adjusted to the desired density. Subsequently, 2 mL of cells at a density of 1 × 10^5^ cells/mL was inoculated into each well of 6-well plates and grown to the appropriate density for uptake experiments. SR solution (Control), SR-SUS, and SR-SUS-ISG, each labeled with coumarin 6 (Cou6) at a concentration of 10 ng/ml, were incubated with the cells for 0.5 h, 1 h, 2 h, and 4 h. For qualitative analysis, after 4 h of incubation, the cells were washed with pre-cooled PBS, fixed by adding 4% paraformaldehyde for 15 min, and then stained by adding DAPI stain for 5 min. The cells were washed with pre-cooled PBS for 2–3 times, and visualized and imaged with a fluorescence inverted microscope (Zeiss, BMI3000, Germany). For quantitative analysis, the cells were rinsed with PBS, and 200 µL of cell lysate was added to each well, followed by lysis on ice for 0.5 h. The lysed cells were then centrifuged at 9000 rpm for 5 min. The supernatant (40 µL) was diluted in 120 µL of methanol and subsequently measured by HPLC at excitation/emission wavelengths of 465/502 nm.

#### Mechanism of cell uptake

HCECs were cultured until reaching 90% confluence, followed by digestion, cell counting, and adjustment of cell densities. For experiments, cells were inoculated into 12-well plates at a density of 1 × 10^5^ cells per well and grown to the desired density. The investigation of the cytosolic pathway involved the addition of specific inhibitors targeting distinct cellular uptake pathways, as outlined in Table 1. HCEC were preincubated with the inhibitors in a 12-well plate for 30 min, after which SR-SUS and SR-SUS-ISG labeled with Cou6 and Cou6 suspension were added and further incubated for up to 4 h. Following the incubation period, cells were rinsed with PBS, and 200 µL of cell lysate was added to each well, followed by a 0.5-hour lysis on ice. Subsequently, 400 µL of methanol was added, and the cells were centrifuged for 5 minutes at 9000 rpm before being quantified by HPLC.

**Table 1:** Inhibitors and their main effects

Inhibitor	Concentration	Primary Action
Chlorpromazine	6 µg/mL	Inhibitor of clathrin-mediated endocytosis
Indomethacin	100 µM	Inhibitor of cell membrane caveolae-like invaginations-mediated endocytosis
Heparin	100 µg/mL	Specific inhibitor of heparan sulfate proteoglycans
Amiloride	10 µM	Specific inhibitor of macrophagocytosis
Sodium azide	0.10%	General inhibitor of endocytosis process
Hypertonic sucrose	0.45 M	Inhibitor of lattice protein-mediated endocytosis through K^+^depleting effects
Methyl-β-cyclodextrin	10 mM	Cholesterol depleting agent capable of inhibiting lipid raft/envelope cellar-dependent cytosis
Chloroquine	125 mM	Destroys endosomes and lysosomes, prevents endosomal acidification, and causes swelling of endosomes and lysosomes
Mycophenolate	10 µg/mL	Inhibits lipid raft/periplasmic caveolae-like invagination-dependent endocytosis via cholesterol chelation

### Animals

All Sprague Dawley (SD) rats (200–250 g, 6–8 weeks) and New Zealand rabbits (2–2.5 kg) were procured from the Experimental Animal Center of Zhejiang Academy of Medical Sciences in Zhejiang, China. The animals were housed under controlled conditions, with a temperature of 19 ± 1 °C and a relative humidity of 50 ± 5%. They were provided with a standard pellet diet and access to water. All animals appeared healthy with no ocular abnormalities.

#### Ethics statement

The whole procedure adhered to the guidelines for animal experiments set forth by the Animal Ethics Committee at Hangzhou Medical College, Huanglong Campus, with the Use License No. SYXK (Zhejiang) 2019-0011. The Ethical Review of Animal Experiments approved this study (Ref. No. 2021 − 163). The animals were sourced from Hangzhou Yuhang Kelian Rabbit Specialized Cooperative. The production of rabbits was conducted under the Production License No. SCXK (Zhejiang) 2017-0004, and the production of the rats was conducted under the Production License No. SCXK (Zhejiang) 2019-0002.

#### Safety evaluation

Healthy New Zealand rabbits were divided into two groups: Saline and SR-SUS-ISG. Each eye received one drop of the formulation (approximately 50 µL) twice a day for a continuous period of 7 days. Throughout the experiment, photos were captured on days 1, 2, 3, 4, 5, 6, and 7 to document the condition of the cornea and conjunctiva. At the conclusion of the study, the rabbits were humanely euthanized by intravenously injecting air needles at the ear margin. Subsequently, the eyeballs were carefully removed and subjected to section hematoxylin-eosin (H&E) staining for histological analysis.

#### Ocular surface retention test

New Zealand rabbits were divided into three distinct groups: the Control group receiving FITC solution (0.2 mg/mL) and two experimental groups treated with the same concentration of FITC-labeled SR-SUS and SR-SUS-ISG. To induce anesthesia, a 20% urethane solution was administered through intravenous injection at the ear margin, with a dosage of 1000 mg/Kg. Subsequently, 50 µL of the respective formulations were instilled into each rabbit’s eye. The eyes were exposed to intervals of blue light from a slit lamp (Shinova Medica, SL-PA, China) and photographed for record-tracking purposes.

#### Pharmacokinetic studies of rabbit eye aqueous humor

##### Pharmacokinetic methods

Fourteen healthy New Zealand rabbits were selected and securely positioned vertically using a brain stereotaxic apparatus, with seven rabbits assigned to each group (SR-SUS and SR-SUS-ISG). Anesthesia was induced with a 20% urethane solution at a dose of 1000 mg/Kg. Tropicamide was employed to dilate the pupils of the rabbits’ eyes. A 25G syringe needle, cut to a length of 5 mm, was affixed to a thin flexible tube using tissue glue. For aqueous humor collection, the rabbit cornea was punctured, and the opposite end of the flexible tube was clamped with a medium-sized arterial clip. The cornea was also affixed to the needle using tissue glue. Excess tear fluid was drained with a cotton swab, allowing time for aqueous humor recovery. Following this, a 50 µL formulation was administered, and 30–40 µL of aqueous humor was collected at intervals of 0.5, 1, 2, 3, 4, 5, 6, 7, 8, 9, and 10 h. The collected samples were stored at -20 ℃. For analysis, a volume of 20 µL from each sample was transferred to a 1.5 mL EP tube, to which 60 µL of the internal standard was added. The mixture underwent vortexing and shaking for 5 min, followed by centrifugation at 4 ℃, 15,000 rpm for 10 min. The supernatant was collected and subjected to LC-MS/MS analysis.

##### LC-MS/MS conditions

The LC-MS/MS system consisted of a HPLC system (Shimadzu, LC-20 A, Japan) and a tandem mass spectrometer (AB Sciex, API4000, USA).

Liquid phase conditions: A Phenomenex Luna 5 μm Phenyl-Hexyl 100 A column measuring 50 × 2 mm was utilized, with mobile phases consisting of 10 mM ammonium formate (A) and methanol (B) employing a gradient elution method as follows: 0 min, A:B = 90:10 (v/v); 0.50 min, A:B = 5:95 (v/v); 4.10 min, A:B = 90:10 (v/v); 5 min, stop. The flow rate was maintained at 0.7 mL/min, the injection volume was set to 5 µL, and the column oven temperature was maintained at 40 ℃.

**Fig. 3:**
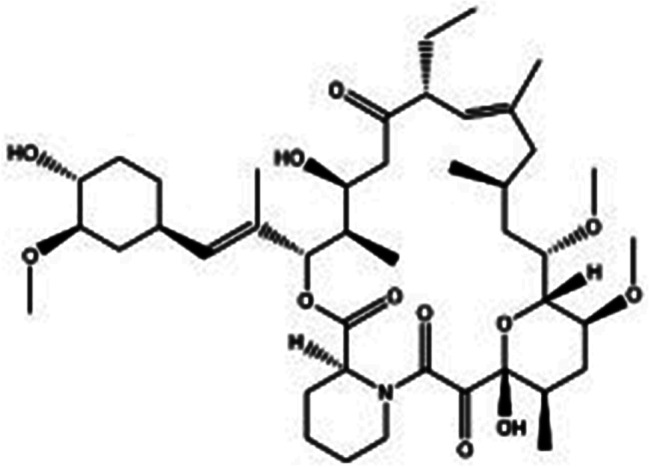
The structural formula of ascomycin [41] (Copyright 2021, Molecules)

Mass spectrometry conditions: Electrospray ionization (ESI) was employed, utilizing the multiple reaction monitoring (MRM) mode. Ascomycin was chosen as the internal standard(IS), as depicted in Fig. 3. The vortex ionization spray temperature was set at 500 ℃, and the electrospray voltage was 5500 V. The detected ion pairs for SR and IS were 931.8→864.7 and 809.8→756.7, respectively. The optimized de-clustering voltage (DP) for positive ion mode was set at 80 V, the injection voltage (EP) was 10 V, and the collision chamber ejection voltage (CXP) was 10 V for both SR and IS. The collision voltage (CE) was set at 19 V for SR and 28 V for IS.

#### Rat alkali burn model

##### Alkali burn model

Rats were anesthetized using 1% pentobarbital at a dose of 40 mg/kg. A 3 mm filter paper soaked in 4 µL of 1 M NaOH solution was placed on the cornea for 30 s. Subsequently, the filter paper was positioned on the central corneal surface for an additional 40 s. The cornea was promptly and thoroughly rinsed with sterile saline for 1 min. Levofloxacin was applied to the eyes three times a day for three days. On the fourth day, rats were divided into groups: Saline, Commercial formulation (cyclosporine eye drops II, CsA), SR-SUS, and SR-SUS-ISG. Each group received 10 µL/dose three times a day for 14 days.

##### Clinical evaluation

Corneal neovascularization in rats was documented using a hand-held slit lamp on days 3, 7, and 14. After 14 days of formulation application, rat corneas were scored based on established criteria [42], as shown in Table 2.

**Table 2:** Scoring criteria for alkaline burns in rats

	Description	Scores
Degree of corneal opacification	Completely clear.	0
	Mildly cloudy, pupil iris easily visible	1
	Mildly cloudy, pupil iris slightly difficult to see	2
	Cloudy, pupil almost invisible	3
	Complete clouding, pupil not visible	4
Length of neovascularization	No blood vessels	0
	Vascularization primarily at the corneal limbus	1
	Neovascularization beyond the corneal limbus and close to the center of the cornea	2
	Blood vessels reach the center of the cornea	3
Neovascularization visibility	No blood vessels	0
	Neovascularization detectable under the slit lamp	1
	Neovascularization easily visible under slit lamp	2
	Neovascularization easily visible without a slit lamp	3

##### H&E staining

Rat eyeballs were fixed in eyeball fixative and embedded in paraffin. Sections were cut, followed by dewaxing and deparaffinization in xylene I and II for 5 min each. Hematoxylin staining was performed for 5 min, followed by a 1-minute 5% acetic acid differentiation, and returning to blue. Eosin staining was carried out for 1 min, followed by dehydration, permeabilization, and sealing. Photomicrographs were captured using a light microscope (Olympus, CX31, Japan).

##### Immunohistochemistry

Rat eyeballs, fixed in eyeball fixative, were paraffin-embedded and sectioned. After deparaffinization, antigenic repair was performed. Incubation with 10% goat serum at room temperature for 30 min preceded overnight incubation at 4 °C with the VEGFA primary antibody. The secondary antibody was then incubated at 37 °C for 45 min. Nuclei were counterstained with hematoxylin, resulting in blue coloration, and observed under the microscope.

##### PCR quantification

Corneas were individually cut, added to 0.6 mL of RNAex (performed on ice), homogenized, and lysed. The lysate was centrifuged in a freezing centrifuge at 4 °C for 5 min at 12,000 rpm, and the supernatant was collected. RNA extraction followed the protocol of the SteadyPure RNA Extraction Kit, and the extracted RNA was stored at -80 °C. Total RNA was reverse transcribed into cDNA using the Evo M-MLV Reverse Transcription Premix Kit. RT-PCR was performed on a fluorescent quantitative PCR instrument (Thermo Fisher, ABI QuantStudio3, USA) with the SYBR^®^ Green Pro Taq HS Premix qPCR Kit.

### Statistical analysis

Data were expressed as the mean ± standard deviation (SD). Statistical analyses were carried out using GraphPad software version 8 and assessed using t-tests or one-way analysis of variance (ANOVA). A *p*-value < 0.05 was statistically significant.

## Results and discussion

### Formulation screening

**Fig. 4:**
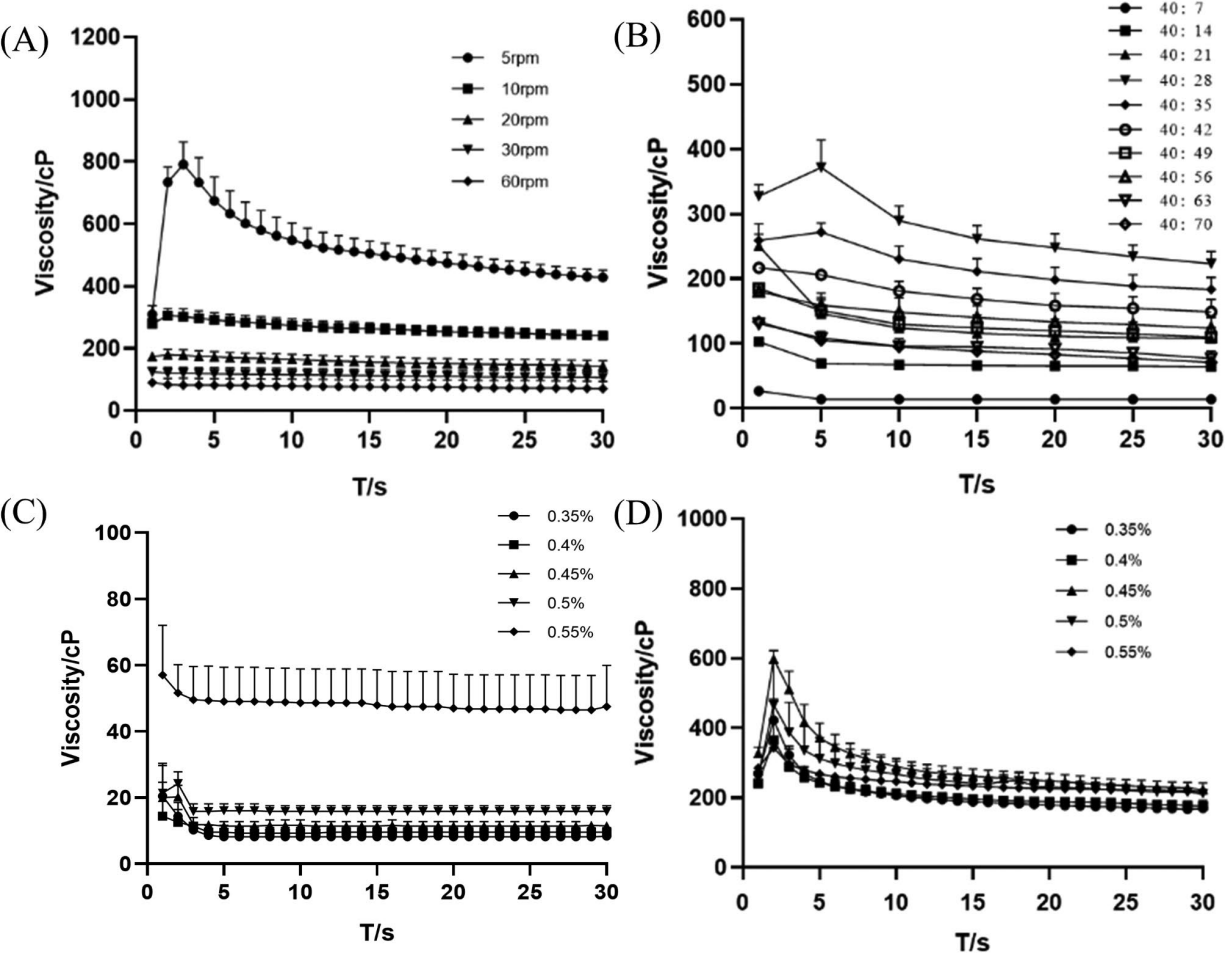
Determination of viscosity of formulations (**A**) Viscosity of sirolimus gel at different rotational speeds (*n* = 3); (**B**) Viscosity of sirolimus gel mixed with artificial tears in different proportions (*n* = 3); (**C**) Viscosity of different concentrations of colloidal preparations at 25 °C (*n* = 3); (**D**) Viscosity of different concentrations of colloidal preparations mixed 40:28 with artificial tears at 34 °C (*n* = 3)

Prescription screening was done by determining the viscosity. According to several studies, the concentration range of gellan gum falls between 0.3% and 0.6%, as concentrations below or above this range may impact its gel-forming ability [43]. In Fig. 4 (A), the concentration of gellan gum mixed with artificial tears at a specific concentration was depicted at 34℃ under varying rotational speeds. Notably, the most suitable rotational speed appeared to be 10 rpm. Due to the properties of gellan gum, phase transition occurred when it was mixed with STF and the viscosity would increase. The proportion of gellan gum remained unchanged, and the viscosity of the mixture would gradually increase as the proportion of STF increased, but after the proportion of STF was raised to a certain level, it would act as a diluent and the viscosity would decrease. Therefore, in order to screen the concentration of gellan gum, the ratio of mixing with STF must first be determined, and then only after mixing different concentrations of gellan gum with it, the optimal prescription can be selected. Figure 4 (B) illustrated the viscosity of gellan gum mixed with artificial tears at different ratios, specifically at 10 rpm and 34 °C. The maximum viscosity was observed when the ratio was 40:28 with artificial tears. In Fig. 4 (C), the viscosity of different concentrations of gellan gum at 25 °C was presented. Notably, the viscosity of 0.55% gellan gum at 25 °C exceeded 40 cP, rendering the formulation overly viscous and inconvenient for canning and transportation. Lower concentrations exhibited better flowability. However, in Fig. 4 (D), among the viscosities measured at 34 °C with artificial tears in a ratio of 40:28 (at maximum viscosity), the viscosity of 0.45% gellan gum concentration was the highest, indicating its potential to linger on the ocular surface for an extended duration. Hence, the optimal concentration was 0.45% gellan gum, showcasing lower viscosity and a liquid state at room temperature and higher viscosity transitioning to a gel state at 34 °C, aligning with the desired properties of in situ gels.

### Characterization of SR-SUS-ISG

#### Particle size, PDI, Zeta potential and content determination

**Fig. 5:**
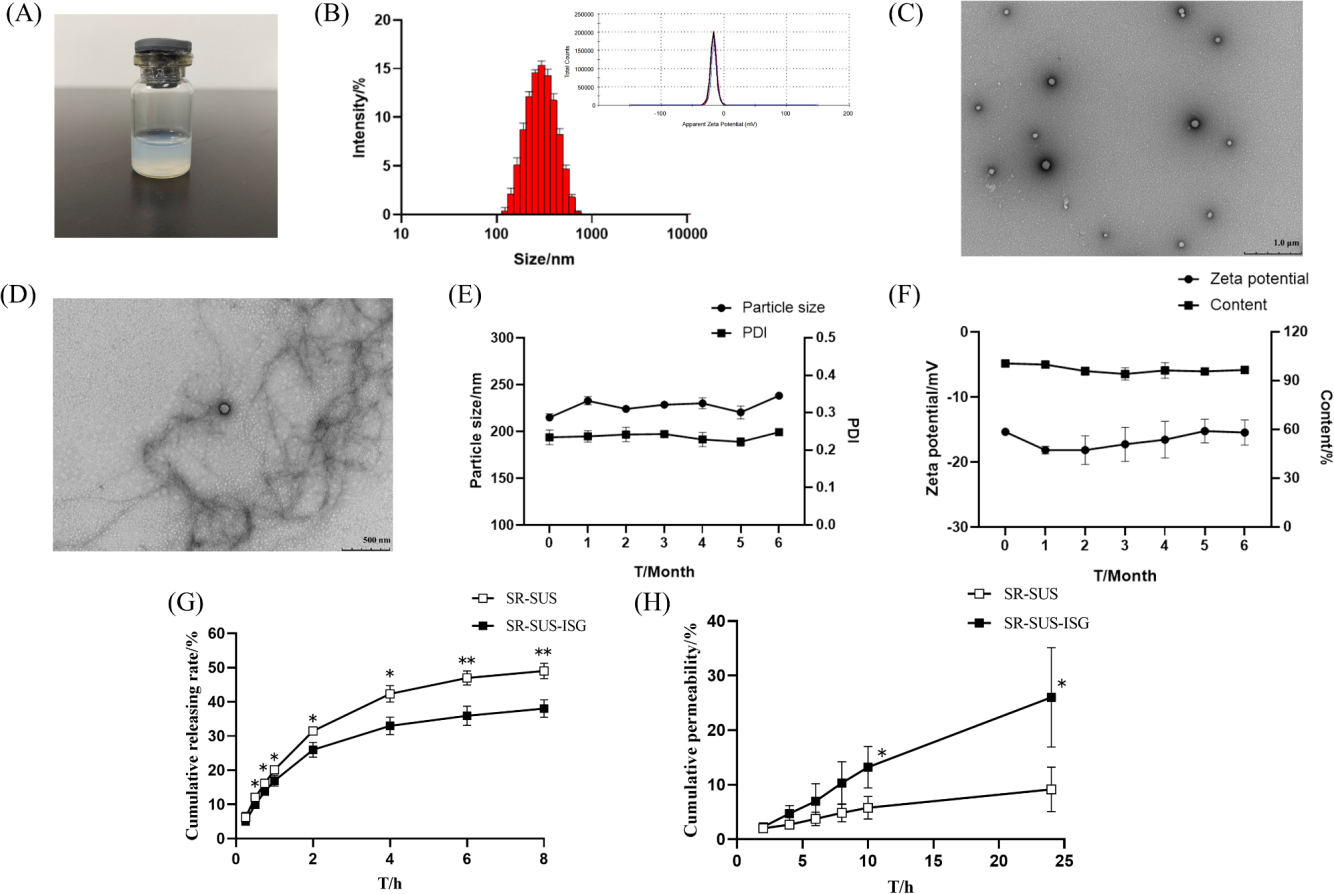
Characterization of SR-SUS-ISG (**A**) Image of SR-SUS-ISG; (**B**) Particle size distribution and Zeta potential (*n* = 3); (**C**) Transmission electron microscopy image of SR-SUS-ISG (Scale bar: 1.0 μm); (**D**) Transmission electron microscopy image of SR-SUS-ISG mixed with artificial tears at 40:7 (Scale bar: 500 nm); (**E**) Changes in particle size and PDI of SR-SUS-ISG in 6 month (*n* = 3); (**F**) Changes in Zeta potential and content of SR-SUS-ISG in 6 month (*n* = 3); (**G**) in vitro release (*n* = 3); (**H**) Scleral permeation (*n* = 3); Data are presented as the mean ± SD, *p < 0.05, **p < 0.01

As seen in Fig. 5 (A), SR-SUS-ISG was uniformly transparent in milky blue. The particle size of SR-SUS-ISG was 228.63 ± 1.59 nm, as depicted in Fig. 5 (B). The size distribution exhibited a single peak, indicating a uniform distribution. The zeta potential was − 16.23 ± 0.80 mV. The PDI was 0.21 ± 0.01, indicating a well-dispersed formulation. The content of SR-SUS-ISG was determined to be 99.34 ± 0.72% (*n* = 6).

#### Transmission electron microscopy

Transmission electron microscopy (TEM) images of SR-SUS-ISG were presented in Fig. 5 (C, D). Figure 5(C) illustrated the distribution of SR-SUS-ISG, revealing spherical particles with a smooth surface. In Fig. 5 (D), SR-SUS-ISG was combined with artificial tear fluid at a ratio of 40:7, where filaments around the spherical shape of SR-SUS-ISG can be observed, suggesting the presence of conjugates of ISG mixed with artificial tears.

#### Stability studies

The stability of SR-SUS-ISG over 6 months at 4 °C was shown in Fig. 5 (E, F). The particle size was in the range of 220 nm ~ 240 nm, the PDI was in the range of 0.22 ~ 0.25, the Zeta potential was in the range of -15 mV~-20 mV, and the content was above 90%. SR-SUS-ISG was stable when stored at 4 °C for 6 in vitro.

#### In vitro release

In this investigation, the release medium consisted of 20% ethanol added to 1% SDS-PBS. Previous studies on posaconazole suspension in situ gel compared release media with and without Tween in artificial tears, revealing that the addition of Tween accelerated release (from 10–25%) [20]. This suggested that the inclusion of solubilizers in the release media can enhance the in vitro release of BCS II drugs. Similarly, for sirolimus, a BCS II drug, studies have shown that adding 20% ethanol to the release medium accelerates the release, as observed in the comparison of SR liposomes with an SR ethanol solution [44]. Before the in vitro release, solubility measurements were performed. The saturated solubility of SR in 1%SDS-PBS was 257.32 µg/mL and the saturated solubility of SR in 20% ethanol-1% SDS-PBS was 407.53 µg/mL. Since the sink condition requires that the volume of the release medium be at least 3 times the volume of the medium needed to saturated dissolve the API in the formulation. In this experiment, 0.5 mL of 1 mg/mL SR-SUS-ISG in 20 mL of release medium for in vitro release was able to satisfy the leaky tank condition. An initial attempt with 1% SDS-PBS as the release medium demonstrated a low cumulative release rate of SR-SUS-ISG over 24 h, approximately 15%. Subsequently, 20% ethanol was added to 1% SDS-PBS, which improved the release conditions. The results presented in Fig. 5 (G) demonstrated a higher cumulative release rate of SR-SUS compared to SR-SUS-ISG, showing a significant difference (*p* < 0.05), which became highly significant (*p* < 0.01) after 6 h of release. The utilization of a dialysis bag with a constant total drug amount, combined with the presence of gellan gum, contributed to a reduced drug release rate in the SR-SUS-ISG formulation. Table 3 provided the fitting results from Origin Software 95 (Origin Software, Inc., OriginLab, USA) for the in vitro release of the two sirolimus formulations. Both formulations conformed to a first-order kinetic process, indicating passive drug transport and a progressively decelerated release over time. The Ritger-Peppas equation suggests that if *n* = 1, the release mechanism is zero-order; if *n* ≤ 0.5, it indicates Fickian diffusion; and values of *n* between 0.5 and 1 suggest a non-Fickian diffusion release mechanism, primarily diffusion and decomposition [45]. For both SR-SUS and SR-SUS-ISG, the calculated *n* values were less than 0.5, indicating that their release mechanisms followed Fickian diffusion.

#### Scleral permeation

The results of ex vivo scleral permeation of SR-SUS and SR-SUS-ISG were presented in Fig. 5 (H). The cumulative permeation of SR-SUS at 24 h was 9.16 ± 4.08%, whereas the cumulative permeation of SR-SUS-ISG at 24 h was 26.00 ± 9.11%, showing a significant difference between the two at 10 h versus 24 h (*p* < 0.05). As indicated in Table 4, the P_app_ of SR-SUS was (0.704 ± 0.330) ×10^− 3^ cm/h, and that of SR-SUS-ISG was (1.929 ± 0.621) ×10^− 3^ cm/h. Given that C_0_ was 1 mg/mL, the steady-state flux (J_ss_) values for SR-SUS and SR-SUS-ISG were 0.704 ± 0.330 µg/cm²/h and 1.929 ± 0.621 µg/cm²/h, respectively. Both P_app_ and J_ss_ of SR-SUS-ISG were 2.73 times higher than those of SR-SUS, exhibiting a significant difference (*p* < 0.05) between SR-SUS-ISG and SR-SUS in both J_ss_ and P_app_. The observed differences could be attributed to the curved opening of Franz’s osmotic cell, allowing rapid loss of SR-SUS with only a small amount of drug remaining on the scleral surface. In contrast, SR-SUS-ISG, when mixed with artificial tears in a ratio of 40:7, exhibited a certain viscosity that prolonged the retention time in the curved opening. Previous studies have suggested that the phase transition of gellan gum may absorb water from the mucosa, leading to transient widening of the tight junctions and enhanced penetration [46]. Based on this research, we were originally intended to conduct ex vivo corneal and ex vivo scleral penetration experiments, while the activity of the cornea may limit the duration of penetration. Previous studies have demonstrated the existence of the tight junction protein claudin-1 in choroidal fibroblasts and retinal pigment epithelium [47, 48]. Hence isolated sclera penetration experiment was chosen for this study. Indeed, scleral penetration experiments were performed after removal of the cornea and intraocular contents, which actually encompassed the sclera, choroid and retina. In this study, the cumulative release rate from isolated sclera was found to be half that of in vitro release. A comparison with a study on ranibizumab liposomes, evaluating in vitro dialysis membrane versus isolated scleral permeation, revealed that when the amount of drug evaluated in both in vitro and isolated experiments was the same, the in vitro dialysis membrane release was nearly 100%, while the isolated scleral permeation was only 50%. This difference might be due to the sclera’s potential to act as a reservoir and provide sustained release from the subconjunctival/suprascleral gap [49].

**Table 3:** Results of in vitro release fitting curve

Models	SR-SUS		SR-SUS-ISG	
	Fitting equation	*R* ^2^	Fitting equation	*R* ^2^
Zero-order	A = 2.229 + 0.859t	0.755	A = 1.779 + 0.909t	0.771
First-order	A = 7.160*(1-e^− 0.685t^)	0.963	A = 37.283*(1-e^− 0.608t^)	0.998
Higuchi	A = 20.235*(t^0.5)-1.097	0.948	A = 15.551*(t^0.5)-0.747	0.940
Ritger-peppas	A = 19.479*(t^0.498)	0.946	A = 14.975*(t^0.498)	0.938

**Table 4:** Calculation results of scleral permeation for P_app_ and J_ss_(*n* = 3, $\overline{x}$±s); *p<0.05

Biofilm	Formulation sample	Papp(cm/h)	Jss(µg/cm^2^/h)
Sclera	SR-SUS	(0.704 ± 0.330)×10^− 3^	0.704 ± 0.330
	SR-SUS-ISG	(1.929 ± 0.621)×10^− 3^*	1.929 ± 0.621*

### Cells

#### Cell proliferation

**Fig. 6:**
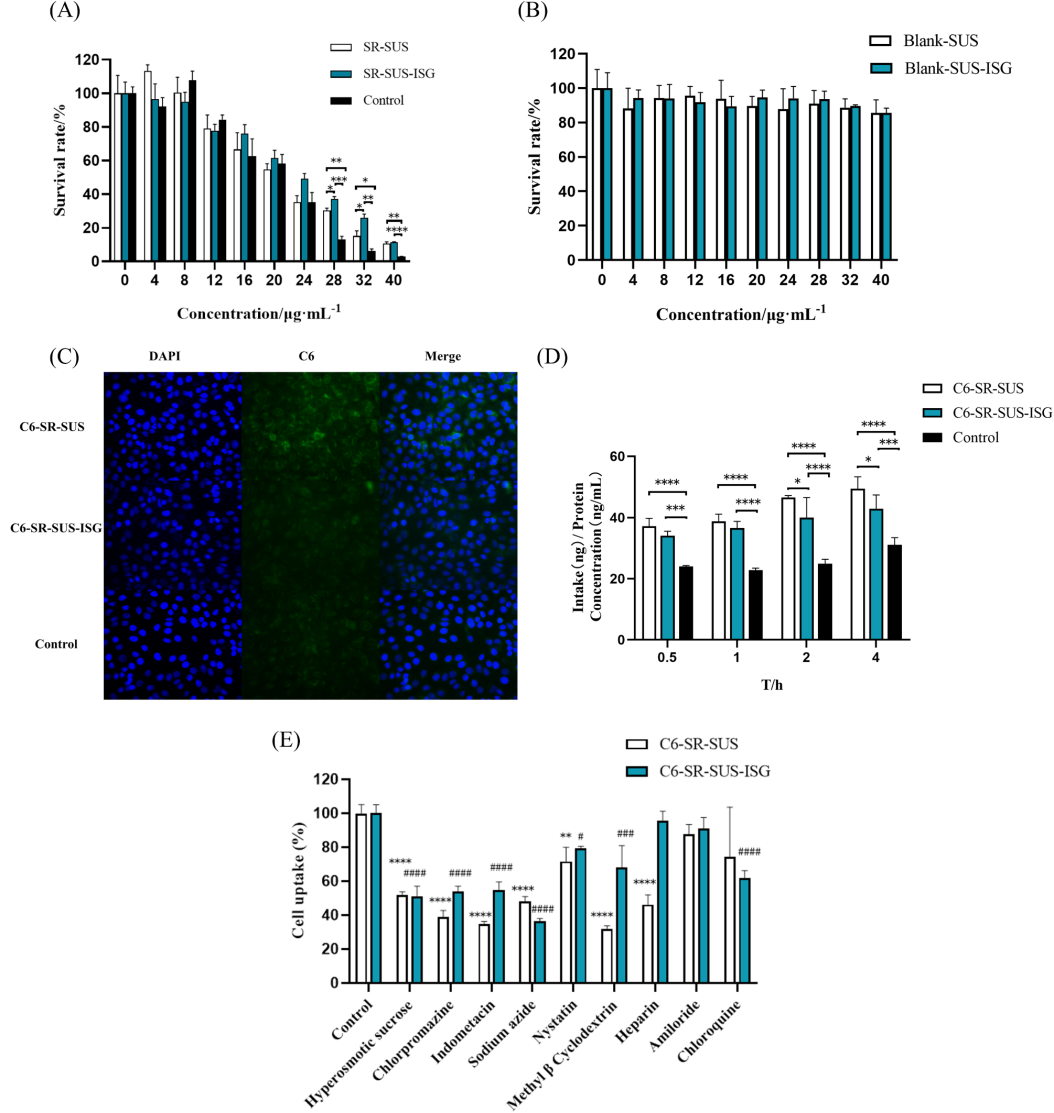
The Cell testing of SR-SUS and SR-SUS-ISG(**A**) CCK8 assay of HCECs exposed to SR Formulations(*n* = 3); (**B**) CCK8 assay of HCECs exposed to Blank Formulations(*n* = 3);(**C**) Uptake image of C6-SR co-loading preparation by HECE cells at 4 h ; (**D**) Uptake assay of nano-preparations of SR co-loaded with Cou6 by HECE(*n* = 3); (**E**)The uptake mechanism of nano-preparations of SR co-loaded with Cou6 by HECE(*n* = 3); C6-SR-SUS: *p < 0.05,**p < 0.01,***p < 0.001,****p < 0.0001C6-SR-SUS-ISG: ^#^*p* < 0.05, ^##^*p* < 0.01, ^###^*p* < 0.001, ^####^*p* < 0.0001; Data are presented as the mean ± SD.

In Fig. 6 (A), the toxicity results of SR-loaded formulations on HCECs at 24 h were depicted. The cell viability gradually decreased with the increase in SR concentration. The cell viability of both SR formulations was higher than that of the SR solution (Control group). When the SR concentration exceeded 28 µg/mL, the cell viability of SR-SUS-ISG was significantly higher than that of SR-SUS and the Control, indicating that both SR-SUS and SR-SUS-ISG had a mitigating effect on toxicity. Figure 6 (B) illustrated the toxicity results of SR blank preparations on HCECs at 24 h. The HCEC viability for both SR blank preparations was above 80%, indicating that the blank preparation excipients were biocompatible.

#### Cellular uptake

In Fig. 6 (C), the uptake image of C6-SR co-loading formulations by HECE cells at 4 h was shown. There were differences in the intensity of green fluorescence among the groups, with the C6-SR-SUS group showing the highest green fluorescence, which was significantly stronger than that of the other two groups. The intensity of green fluorescence in the C6-SR-SUS-ISG group was higher than that in the Control group. In Fig. 6 (D), the uptake of Control, C6-SR-SUS, and C6-SR-SUS-ISG by HCECs at 0.5, 1, 2, and 4 h was presented. The uptake of Cou6 by HCECs gradually increased with the extension of time, indicating time-dependent uptake of Cou6 by HCECs. Among them, the uptake of Cou6 and SR co-loading preparation by HCECs at 0.5 h, 1 h, 2 h, and 4 h was higher than that of the Control group, respectively, with significant differences (*p* < 0.001). Furthermore, the uptake of C6-SR-SUS was significantly higher than C6-SR-SUS-ISG at 2 h and 4 h (*p* < 0.05). The physical state of the formulation was a less viscous gel, and thus cellular uptake of SR-SUS-ISG was more difficult than SR-SUS. Besides, the particle size of SR-SUS was 131.2 ± 0.88 nm, while the particle size of SR-SUS-ISG was 228.63 ± 1.59 nm. The uptake of C6-SR-SUS by HCECs was higher than that of C6-SR-SUS-ISG, possibly due to the particle size . This size difference has been suggested as a contributing factor, as smaller nanoparticle sizes are considered the most important factor controlling cellular uptake of nanoparticles [50].

#### Mechanisms of Uptake

Figure 6 (E) illustrated the results of mechanistic studies on the uptake of HCECs by C6-SR-SUS and C6-SR-SUS-ISG. The uptake of SR by HCECs involved various mechanisms independent of amiloride-mediated enhancement effects on specific macrophages. Among these mechanisms, hypertonic sucrose, chlorpromazine, indomethacin, sodium azide, and methyl-β-cyclodextrin significantly inhibited the cellular uptake of C6-SR preparations (*p* < 0.001). This uptake was mediated by K^+^ depletion effects, lattice proteins, lipid raft/envelope cell-dependent phagocytosis, and caveolae-like invaginations of the cell membrane. Mycobacteriocin inhibited C6-SR-SUS uptake (*p* < 0.01), and the pathway of inhibition was related to the inhibition of lipid raft/envelope caveolae-like endocytosis via cholesterol chelation. Mycophenolic acid inhibited SR-SUS-ISG uptake through the same effect (*p* < 0.05). Heparin significantly inhibited C6-SR-SUS uptake (*p* < 0.001), and the mechanism of uptake was related to heparan sulfate proteoglycans (HSPGs). Additionally, chloroquine inhibited C6-SR-SUS-ISG uptake by disrupting endosomes and lysosomes, preventing endosomal acidification, and causing swelling of endosomes and lysosomes (*p* < 0.001). The uptake of SR-SUS-ISG was lower than that of SR-SUS, in addition to particle size acting as an impact on uptake, it was also possible that the uptake of heparan sulphate proteoglycans was blocked by the gellan gum, and the degradation of endosomes and lysosomes was increased.

### Animals

#### In vivo safety

**Fig. 7:**
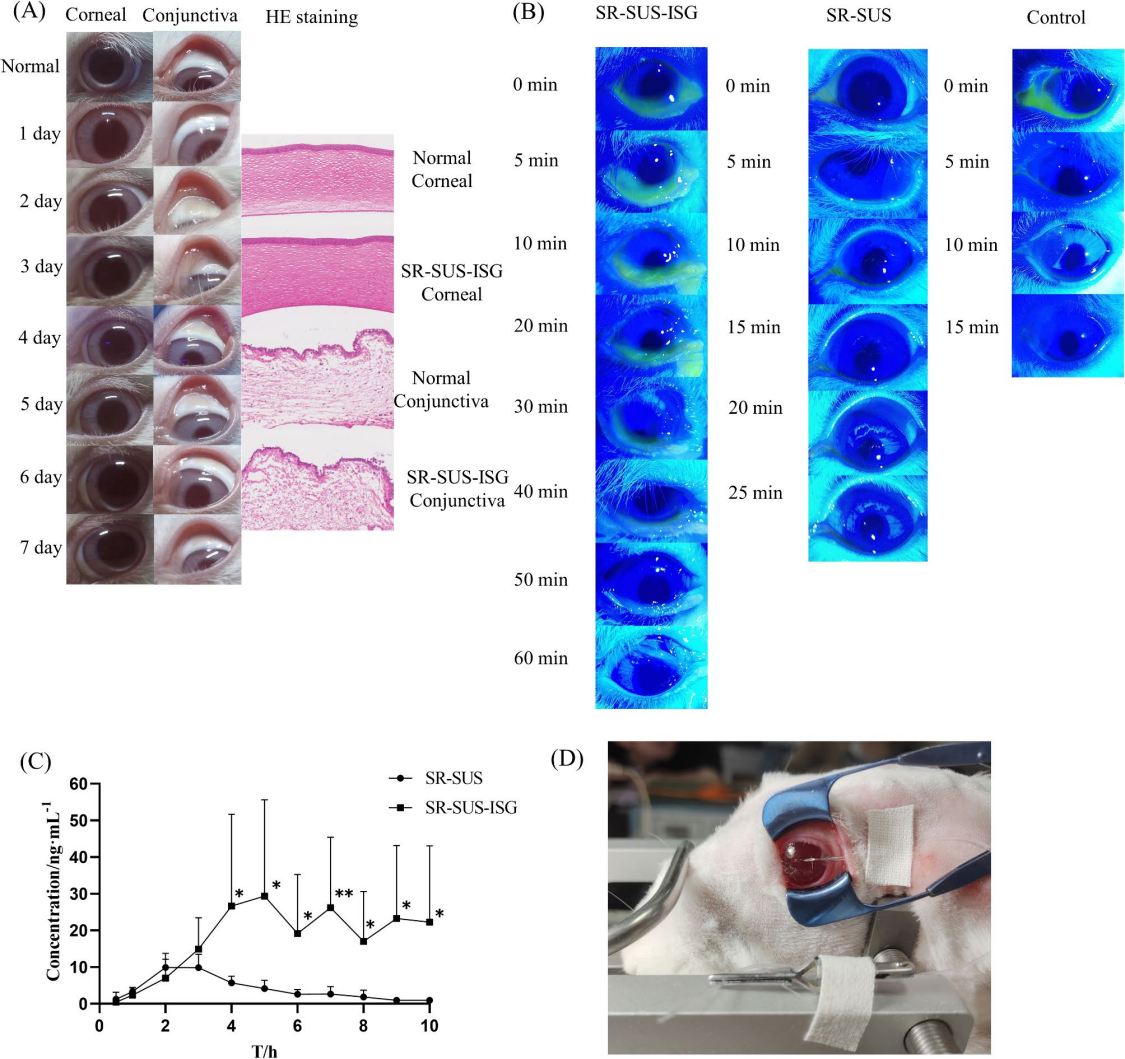
Safety, ocular surface retention and pharmacokinetic studies of aqueous humor in rabbit eyes (**A**) In vivo safety (**B**) Rabbit ocular surface retention (**C**) Rabbit aqueous humor pharmacokinetics(*n* = 7) (**D**) Pharmacokinetic test pictures; Data are presented as the mean + SD *p < 0.05, **p < 0.01, ***p < 0.001

As shown in Fig. 7 (A), the cornea, iris, and conjunctiva were clearly visible in both the Saline and SR-SUS-ISG groups, with few clinical signs of ocular irritation, such as redness, inflammation, or swelling of the eyes. No conjunctival discharge, conjunctival edema, or conjunctival redness was observed. Stained sections of rabbit eyes showed that neither tissue structure nor integrity was affected, and none of the tissues showed significant inflammatory cell infiltration or proliferation, edema, or histopathological changes. This indicated that the preparation had good biocompatibility.

#### Ocular surface retention

Figure 7 (B) illustrated the retention phenomenon for the three FITC-labelled formulations on the ocular surface of rabbits. In the Control group, a small amount of formulation residue could be photographed on the ocular surface at 0 min due to the rapid loss of the ocular surface. At 15 min, only a trace of fluorescence was observed at the inferior eyelid. In the SR-SUS group, a small residue was still concentrated at the corner of the eye at 15 min, and a trace of fluorescence was observed at the corner of the eye at 25 min. In the SR-SUS-ISG group, after administration, most of the drug was concentrated in the conjunctival sac and gradually gelled in reaction to the tear fluid. Due to the blinking reflex and tear dilution, the drug gradually diminished and drained through the nasolacrimal duct. The drug of the Control and SR-SUS group disappeared over time. While the gellan gum could delay the disappearance of the drug in the SR-SUS-ISG group, green fluorescence could still be observed at the lower eyelid by the naked eye at 60 min, indicating that SR-SUS-ISG would prolong the retention time of the drug at the ocular surface.

#### Pharmacokinetics

The drug concentration-time profiles of SR-SUS and SR-SUS-ISG after vertical administration in rabbits were depicted in Fig. 7 (C), while Fig. 7 (D) provided an experimental illustration of the pharmacokinetics of rabbit eye aqueous humor. Pharmacokinetic parameters were summarized in Table 5. The drug concentration remained consistent at 17 ng/mL after four hours, indicating sustained release from 4 h to 10 h. Concentrations in the ocular aqueous humor were significantly higher for SR-SUS-ISG than for SR-SUS (*p* < 0.05). The C_max_ of SR-SUS-ISG was 33.42 ± 26.20 ng/mL, representing a 2.81-times increase compared to SR-SUS (11.88 ± 3.78 ng/mL), and the AUC_(0−t)_ of SR-SUS-ISG was 174.88 ± 124.58 µg/L·h, significantly higher than that of SR-SUS (41.31 ± 12.20 µg/L·h) and 3.54 times higher. The SR-SUS formulation, containing PVA and HPMC, exhibited an affinity for the ocular surface mucosa. The inclusion of ISG, which further increased the viscosity of the drug, resulted in enhanced drug penetration. Notably, the target one-day dose of SR was reported to be 2.6 to 5.2 ng/day [51]. Equally as an immunosuppressant, a study measured the drug concentration in the aqueous humor within 90 min of tacrolimus liposomes and commercially available preparations, and the tacrolimus level was only below 3 ng/mL [52], the present study demonstrated a significantly higher drug concentration in the aqueous humor close to 10 ng/mL at 2 h. This suggested improved penetration of SR into the cornea, indicating that SR-SUS-ISG provided sustained drug release.

**Table 5:** Pharmacokinetic parameters of sirolimus nanosuspension and sirolimus nanogel after vertical administration in rabbits(*n* = 7, $\stackrel{-}{x}\pm$s)*p<0.05

Pharmacokinetic Parameters	SR-SUS	SR-SUS-ISG	Ratio ISG/SUS
AUC_(0−t)_/ µg/L·h	41.31 ± 12.20	174.88 ± 124.58*	4.23
AUC_(0−∞)_/ µg/L·h	43.99 ± 14.43	686.17 ± 1201.20	15.59
MRT_(0−t)_/h	3.81 ± 0.52	5.81 ± 0.49*	-
T_max_/h	0.36 ± 0.10	0.23 ± 0.14	-
C_max_/ng/mL	11.88 ± 3.78	33.42 ± 26.20	2.81

#### Rat corneal neovascularization

##### Observation and scoring after alkali burns

**Fig. 8:**
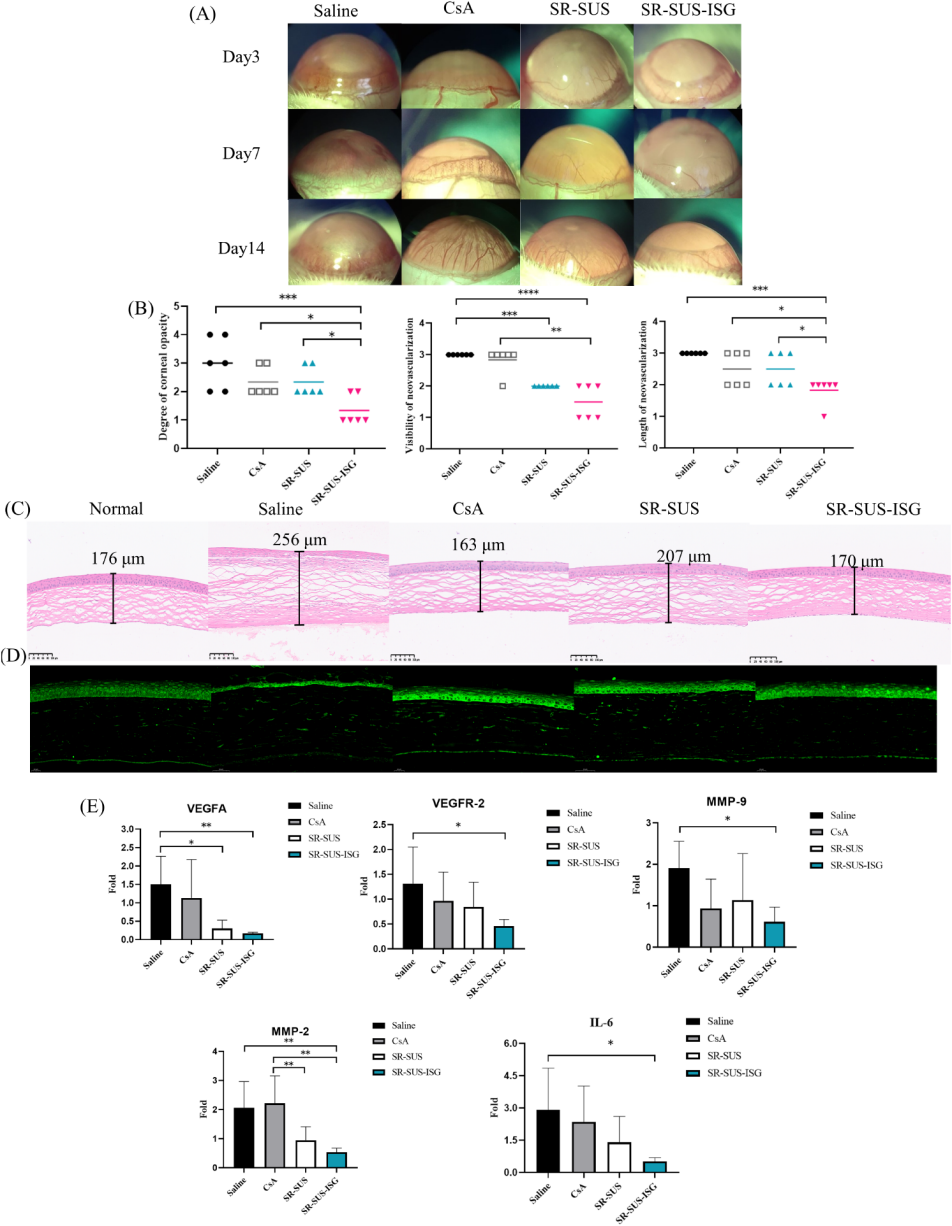
Pharmacodynamic studies (**A**) Photographic recordings of rat corneas with alkali burns on days 3, 7, and 14 (**B**) Rat corneal alkali burn scores (*n* = 6) (**C**) H&E staining of rat corneas (**D**) Immunofluorescence staining of rat corneas with VEGFA (**E**) PCR results (*n* = 6); Data are presented as the mean ± SD *p < 0.05, **p < 0.01, ***p < 0.001

Corneal neovascularization in rats after 14 days of drug administration was depicted in Fig. 8 (A). The corresponding corneal neovascularization scores were presented in Fig. 8 (B). In the Saline group, the eyes of rats exhibited congestion and edema, with a turbid cornea and nearly full neovascularization within 14 days. The CsA and SR-SUS groups showed no edema or congestion, and the cornea was mildly turbid, with dense neovascularization within 14 days. The length of neovascularization extended between the center and 2/3 of the cornea, indicating lower inhibition of corneal neovascularization in the CsA and SR-SUS groups. In contrast, the SR-SUS-ISG group showed no edema or congestion, a clear cornea, and less neovascularization compared to the other three groups. The length of neovascularization was also shorter in the SR-SUS-ISG group. In terms of corneal turbidity scores, there was a significant difference in the SR-SUS-ISG group compared to the first three groups (*p* < 0.05). Regarding corneal neovascularization visibility, a significant difference was observed in the SR-SUS-ISG group compared with the Saline and CsA groups (*p* < 0.001) and the SR-SUS group compared with the Saline group (*p* < 0.01). Additionally, in corneal neovascularization length, SR-SUS-ISG exhibited a significant difference compared to the first three groups (*p* < 0.05).

##### H&E staining

Figure 8 (C) displayed the H&E-stained sections of the cornea in each group after 14 days of drug administration. In the Normal group, the cornea exhibited the typical structure of normal rats, with tightly arranged corneal epithelial cells. Rats in the Saline group showed congested and edematous eyeballs, consistent with the photographic results. The cornea in the Saline group displayed thickening, thinning of the corneal epithelium, crumpling of epithelial cells, and a sparse stromal layer. Extensive neovascularization and inflammatory cells were observed. In the CsA and SR-SUS groups, due to the shorter retention time and reduced infiltration, the corneal epithelium showed wrinkling, the corneal stroma was loose, and neovascularization and inflammatory cells were present. The SR-SUS group exhibited mild thickening. Remarkably, in the SR-SUS-ISG group, there was no corneal thickening, no shrinkage of epithelial cells, and the stroma appeared tightly arranged, with minimal neovascularization. This structure closely resembled that of the normal rat cornea. These findings suggest that SR-SUS-ISG has a positive impact on maintaining the normal corneal structure compared to other formulations.

##### VEGFA immunofluorescence histochemistry

Figure 8 (D) depicted the VEGFA fluorescence-stained sections of the rat cornea in each group after 14 days of administration. In the Normal group, the stromal layer showed less green fluorescence of VEGFA, which was more dispersed. Conversely, the Saline group exhibited more green fluorescence of VEGFA in the stromal layer. In the CsA and SR-SUS groups, the corneal epithelium showed enhanced fluorescence, and there was a higher distribution of green fluorescence of VEGFA in the stroma layer. Remarkably, the SR-SUS-ISG group demonstrated less green fluorescence distribution in the corneal stroma layer, resembling the normal cornea. This suggested that SR-SUS-ISG effectively inhibited VEGFA protein expression in corneal neovascularization compared to other formulations, indicating its potential to suppress angiogenesis-related factors.

##### PCR results

VEGF is a specific marker protein for vascular endothelial cells and plays a crucial role in the regulation of capillary angiogenesis. In this respect, VEGF induces vascular endothelial cell proliferation, chemokine response, and permeability, and it is involved in neointima formation [53]. VEGF has been reported to be a potent factor in the initiation and regulation of angiogenesis [54]. Among them, VEGFA and VEGFR-2 reportedly play an essential position in corneal neovascularization [52]. The results presented in Fig. 8 (E) indicated that SR-SUS-ISG significantly inhibited the expression of VEGFA (*p* < 0.01) and VEGFR-2 (*p* < 0.05) compared to the Saline group, as well as VEGFA in the SR-SUS group (*p* < 0.05). This suggested that SR-SUS-ISG effectively suppresses the expression of genes related to VEGF, a key regulator of angiogenesis. MMPs are matrix metalloproteinases exhibit a dual effect on the described process [55], playing a crucial role in modulating the balance between angiogenic and antiangiogenic factors [56]. Specifically, their protein hydrolysis activity promotes corneal angiogenesis, with MMP-2 and MMP-9 being key components of the vascular basement membrane [57]. In the present study, SR-SUS-ISG demonstrated significant inhibition of MMP-2 (*p* < 0.01) and MMP-9 (*p* < 0.05) expression compared to the Saline group while downregulating MMP-2 expression (*p* < 0.01) in both SR-SUS and SR-SUS-ISG groups compared to the CsA group. These findings suggested that SR-SUS-ISG effectively suppressesed the expression of MMP-related genes. Furthermore, inflammation is a notable characteristic of neovascularization [58]. Research has indicated that systemic and local administration of SR can mitigate corneal opacity and neovascularization induced by alkaline burns in mice by reducing IL-6 levels [59]. In the current investigation, SR-SUS-ISG significantly inhibited the expression of IL-6 (*p* < 0.05) compared with the Saline group. Collectively, these results implied that SR-SUS-ISG may hinder corneal neovascularization by modulating VEGF, MMPs, inflammatory genes, and other related genetic factors.

## Conclusion

In this investigation, SR-SUS-ISG was successfully developed using gellan gum, addressing the challenge of the low solubility of SR and extending its retention time in the eye. Comparative analysis with SR-SUS revealed that SR-SUS-ISG exhibited higher osmotic and steady-state fluxes during ex vivo scleral release studies. Both in vivo and ex vivo assessments confirmed the excellent biocompatibility and enhanced ocular surface retention of SR-SUS-ISG. Pharmacokinetic studies in rabbit aqueous humor demonstrated sustained drug release from SR-SUS-ISG, sustaining therapeutic levels for up to a one-day dosage. Furthermore, SR-SUS-ISG proved effective in inhibiting corneal neovascularization, with the vascular inhibition pathway linked to key genes such as VEGF and MMPs. The overall findings suggest that SR-SUS-ISG holds promise as an ophthalmic formulation and represents a potential candidate for addressing corneal neovascularization in future applications.

## Data Availability

All data generated or analyzed during this study are included in this published article.
